# Determinants of neonatal mortality in Nigeria: evidence from the 2008 demographic and health survey

**DOI:** 10.1186/1471-2458-14-521

**Published:** 2014-05-29

**Authors:** Osita Kingsley Ezeh, Kingsley Emwinyore Agho, Michael John Dibley, John Hall, Andrew Nicholas Page

**Affiliations:** 1 School of Science and Health, University of Western Sydney, Locked Bag 1797, Penrith, New South Wales (NSW) 2571, Australia; 2 Sydney School of Public Health, Edward Ford Building (A27), University of Sydney, Sydney, NSW 2006, Australia; 3 School of Medicine and Public Health, Faculty of Health, University of Newcastle, Callaghan, NSW 2308, Australia

**Keywords:** Determinants, Neonatal mortality, Cox regression, Nigeria

## Abstract

**Background:**

Nigeria continues to have one of the highest rates of neonatal deaths in Africa. This study aimed to identify risk factors associated with neonatal death in Nigeria using the 2008 Nigeria Demographic and Health Survey (NDHS).

**Methods:**

Neonatal deaths of all singleton live-born infants between 2003 and 2008 were extracted from the 2008 NDHS. The 2008 NDHS was a multi-stage cluster sample survey of 36,298 households. Of these households, survival information of 27,147 singleton live-borns was obtained, including 996 cases of neonatal mortality. The risk of death was adjusted for confounders relating to individual, household, and community level factors using Cox regression.

**Results:**

Multivariable analyses indicated that a higher birth order of newborns with a short birth interval ≤ 2 years (hazard ratio [HR] = 2.19, confidence interval [CI]: 1.68–2.84) and newborns with a higher birth order with a longer birth interval > 2 years (HR = 1.36, CI: 1.05–1.78) were significantly associated with neonatal mortality. Other significant factors that affected neonatal deaths included neonates born to mothers younger than 20 years (HR = 4.07, CI: 2.83–5.86), neonates born to mothers residing in rural areas compared with urban residents (HR = 1.26, CI: 1.03–1.55), male neonates (HR = 1.30, CI: 1.12–1.53), mothers who perceived their neonate’s body size to be smaller than the average size (HR = 2.10, CI: 1.77–2.50), and mothers who delivered their neonates by caesarean section (HR = 2.80, CI: 1.84–4.25).

**Conclusions:**

Our study suggests that the Nigerian government needs to invest more in the healthcare system to ensure quality care for women and newborns. Community-based intervention is also required and should focus on child spacing, childbearing at a younger age, and poverty eradication programs, particularly in rural areas, to reduce avoidable neonatal deaths in Nigeria.

## Background

Neonatal mortality is still a significant public health problem worldwide, and accounts for more than 60% of newborn deaths before their first birthday [1]. Of the world’s 7.7 million deaths in those aged younger than 5 years, 3.1 million occurred after birth through to 1 month of life (neonatal deaths) [2]. Nearly 99% of these neonatal deaths occur in low- and middle-income countries, mostly in sub-Saharan Africa, including Nigeria [3]. The majority of these deaths are caused by preventable or treatable diseases, such as infectious diseases, which contribute to approximately 36% of these deaths [3]. Previous studies have shown that the global decline in neonatal mortality rates has been slower compared with infant and under-5 years of age mortality rates, especially in the sub-Sahara African region [1,2,4].

Globally, Nigeria ranks second to India with the highest number of neonatal deaths, with the highest reported number in Africa [5]. Each year in Nigeria, more than a quarter million neonates die, which translates to approximately 700 neonates every day [5]. Neonatal mortality remains disturbingly high in Nigeria, despite the significant decline in most parts of the developing world, including some sub-Sahara African countries, such as Ghana and Uganda [6]. A recent United Nations (UN) report on childhood mortality reported that over the last 2 decades, the Nigerian neonatal mortality rate (NMR) dropped by only 20.4%, from 49 deaths per 1000 live births in 1990 to 39 in 2011 [5]. Similarly, evidence from the Nigeria Demographic and Health Survey (NDHS) also indicated a marginal decline of 4.8% (42 deaths per 1000 live births in 1990 to 40 in 2008) [7]. The 39 and 40 neonatal deaths per 1000 live births reported by the UN and NDHS, respectively, can be interpreted as approximately one in every 25 neonates born in Nigeria died in the first month of life.

Previous studies on neonatal mortality in Nigeria have indicated that low birth weight, lack of antenatal care, maternal illness, mother’s age, prematurity, and birth asphyxia are linked with neonatal mortality, but these studies were all hospital-based case–control and experimental studies [8-11]. Limitations of these hospital-based case–control and experimental studies are that neonates delivered at home were not included and that control groups were not population based, and may not be generalizable to the wider Nigerian population. Evidence from the NDHS showed that home delivery in Nigeria remains high. An example of this situation is in the 1999 NDHS, where approximately 58% of neonates were delivered at home [12], and this number rose to 66% in the 2003 NDHS [13], and was 62% in the 2008 NDHS [7]. Importantly, neonatal mortality rates play an increasingly important role in childhood mortality, and there are currently no effective community based intervention programs in Nigeria specifically targeting neonatal mortality.

The main goal of this study was to determine factors associated with neonatal mortality using the 2008 NDHS. Findings from the study would be useful to public health researchers and policy makers in reviewing and designing new community based intervention strategies aimed at reducing neonatal mortality in Nigeria. Therefore, this study presents population-based data on risk factors associated with neonatal mortality in Nigeria.

## Methods

This study was based on a public domain dataset that is freely available online. The data were collected for the NDHS 2008 [7]. The survey was conducted by the National Population Commission in conjunction with the ICF Macro, Calverton, MD, USA, in 36 states and the federal capital territory [7].

The 2008 NDHS was a stratified two-stage cluster design. Each state was stratified into two distinct groups of urban and rural areas. The census enumeration areas of the 2006 population census were used as the clusters for the 2008 NDHS. In the first stage, clusters were selected based on probability proportionate to the population size among its urban and rural areas. In each of the selected clusters, a complete listing of households was obtained. The listed households then served as the sampling frame for the selection of households to be interviewed in the second stage. Thereafter, a systematic sampling with equal probability was used in the second stage in selecting the specified number of households in each cluster for interview [7].

A structured questionnaire was used for interviewing the selected households for the 2008 NDHS. The questionnaires that were administered to the respondent household members were the household questionnaire, the women’s questionnaire, and the men’s questionnaire. These questionnaires consisted of a series of questions on population and health issues. The household questionnaire recorded all of the usual residents of the selected household and their characteristics, such as age, sex, education, and their relationship with the head of the household, as well as information on amenities and features of the household’s dwelling unit. Additionally, the survey collected data on height and weight measurements for children aged younger than 5 years, and women aged 15–49 years. The women’s questionnaire consists of information included, but not limited to, birth history, childhood mortality, fertility preferences, knowledge and use of family planning methods, antenatal care, delivery, postnatal care, vaccinations, and childhood illnesses, as well as malaria prevention and treatment. The 2008 NDHS men’s questionnaire was the same as the women’s questionnaire, but did not contain a detailed reproductive history, maternal and child health, or nutrition. However, notably, gestational age, intrapartum-related complications, and birth asphyxia, which could potentially improve neonatal data, were not collected in the 2008 NDHS.

A total of 888 clusters were selected for the 2008 NDHS sample survey. Of these clusters, a total of 36,298 households were selected for interview in the 2008 NDHS. At the time of the survey, nearly 5% of the households were not occupied. However, more than 98% of the occupied households were successfully interviewed. A total of 34,596 eligible women aged between 15 and 49 years were interviewed, yielding a response rate of 96.5%. The analysis was restricted to all singleton live births for a 5-year period preceding the 2008 NDHS to reduce recall bias about birth and death dates reported by mothers.

### Descriptive study variables

A conceptual framework of child survival in developed and developing countries has been developed by other authors [14-17]. However, the model by Moseley [15] is regarded as the most elaborate and systematic conceptual framework [18], and is frequently referenced in other studies on childhood mortality [19]. As a result, our study used the Moseley [15] conceptual framework as the basis for identifying important risk factors for neonatal mortality in Nigeria.

The outcome variable for this study was neonatal death as reported by the mothers who participated in the survey, and it was defined as the death of a neonate between birth and 1 month of life. This takes a binary form, such that neonatal death will be regarded as a success (1 = if death occurs in the specified age period) or failure (0 = if the newborn is alive in the specified age period). The outcome variable was examined against all confounding variables, and these variables were classified into three distinct groups: community level factors, household factors, and individual level factors consisting of socioeconomic factors (Table 1). These variables were used in the study to identify risk factors associated with neonatal mortality. Based on the adapted conceptual framework, all of the confounding variables influencing neonatal mortality along with their categorisations are shown in Table 1.

**Table 1:** Definition and categorisation of potential variables used in identifying risk factors associated with neonatal mortality

**POTENTIAL VARIABLES**	**CATEGORISATION**
**Community level factors**	
Residence	Type of the residence (1 = urban; 2 = rural)
Geopolitical zone	Zone (1 = North Central; 2 = North East; 3 = North West; 4 = South East; 5 = South West; 6 = South South)
**Household factor**	
Wealth index	Wealth (1 = Poor; 2 = Middle; 3 = Rich)
**Individual level factors**	
Maternal religion	Maternal religion (1 = traditionalist or other; 2 = Islam; 3 = catholic or other Christian)
Maternal working status	Maternal working status (1 = not working; 2 = working)
Maternal BMI	Maternal BMI (1 = BMI >18.5; 2 = BMI ≤18.5)
Maternal age at first child	Age at first birth (1 = < 20; 2 = 20–29; 3 = 30-39; 4 = 40-49)
Maternal age	Mother’s age (1 < 20; 2 = 20-29; 3 = 30–39; 4 = 40–49)
Maternal literacy level	Literacy level (1 = Able to read parts of & whole sentence; 2 = Cannot read at all)
Paternal education	Education status (1 = No education; 2 = Primary; 3 = Secondary or higher)
Maternal education	Education status (1 = No education; 2 = Primary; 3 = Secondary or higher)
Sex	Sex of the neonate (1 = Female; 2 = Male)
Birth order and birth interval	Birth order and birth interval of neonate (1 = Second or third child, interval > 2 years; 2 = First child; 3 = Second or third child, interval ≤ 2 years; 4 = Fourth or higher child, interval > 2 years; 5 = Fourth or higher child, interval ≤2 years)
Birth place	Place of delivery of the neonate (1 = Home; 2 = Health facility)
Mother’s perceived baby size	Maternal assessment of the neonate size at birth (1 = Average or Larger; 2 = Small or very small)
Antenatal care	Antenatal care received by mother’s (1 = Yes; 2 = No)
Mode of delivery	Mode of delivery (1 = non-caesarean; 2 = caesarean section)
Delivery assistance	Birth attendant during delivery (1 = Health professional; 2 = non-Health professional)
Desire for pregnancy	Mother’s desire for baby (1 = Wanted then; 2 = Wanted later; 3 = Wanted no more)
Postnatal care	Postnatal care of mother’s after birth (1 = Yes; 2 = No)

There were two community level factors used, residence type and geopolitical zone, while the wealth index variable measured the economic status of the household. The wealth index variable was constructed using household facilities and assets, which were weighted, using a principal components analysis [20]. The range of assets considered were a television, radio, and fridge, and ownership of a car, bicycle, and motorcycle. Household facilities were also included, such as the source of drinking water, type of toilet, electricity, and type of building materials used in the place of dwelling. Among the individual factors, there were 14 variables of maternal and child characteristics (Table 1).

In this analysis, two perinatal healthcare variables, antenatal care and postnatal care, were not included because nearly one third of the information was missing. Additionally, we did not include birth weight of neonates because almost half of the neonates were not weighed at the time of birth. However, perceived newborn size at birth by mothers (small or very small, and average or large) was used instead of birth weight because a previous study showed that there is a close relationship between mean birth weight and perceived newborn size by the mother [21].

### Statistical analysis

The NMR was calculated by using a similar method described by Rutstien and Rojas [22]. The crude hazard ratios (HRs) for factors associated with neonatal death were determined by univariate analyses, which were performed using a Cox proportional hazards regression model. In addition, multivariable analysis was used to examine the association between the potential independent variables and the study outcome. Analyses were performed using STATA/MP version 12.0 (StataCorp, College Station, TX, USA). Cox proportional hazards models were fitted using STATA survey commands to adjust for the cluster sampling design, weights, and the calculation of standard errors.

The multivariable analysis models conducted used a stepwise backwards elimination procedure to identify independent variables that were significantly associated with the study outcome. To avoid any statistical bias, we double checked our backward elimination method by using the following procedures: (1) we entered only potential risk factors with a p value < 0.20 obtained in the univariable analysis for backward elimination process, (2) we tested the backward elimination by including all of the variables (all potential risk factors), and (3) we tested and reported any collinearity in the final model. HRs and 95% confidence intervals (CIs) were calculated to assess the adjusted risk factors that affect study outcome, and those with p < 0.05 were retained in the final model.

## Results

Table 2 shows the number of live births, the number of neonatal deaths and NMR by community, the household wealth index, and individual level factors. A weighted total of 27,147 singleton live births of children aged younger than 5 years occurred within the 5-year period preceding the 2008 NDHS, of which the total neonatal deaths over this period was 996 (Table 2). Neonates born to mothers residing in rural residences had a higher NMR than those living in urban residences (NMR: 38.9 vs 31.3). The NMR for neonates born to mothers in poor households was higher than that in mothers in middle-class households (NMR: 39.3 vs 35.6). Neonates whose mothers perceived them as small or a smaller size, had a greater NMR than those of average or larger size (NMR: 57.0 vs 30.0). The majority of live-born neonates were not weighed at birth, and more than half of the neonatal deaths occurred at home.

**Table 2:** Neonatal mortality rates (NMR) with 95% confidence interval (CI)

**Covariates**	**Total live births**	**Neonatal deaths**	**NMR [95% CI]**
***Community level factors***			
**Residence type**			
Urban	8070	253	31.3 (29.2- 33.4)
Rural	19077	743	38.9 (36.6- 41.3)
**Geopolitical zone**			
North Central	3693	122	32.6 (30.6- 34.9)
North East	4452	176	39.6 (37.2- 42.0)
North West	8529	310	35.7 (33.5- 37.9)
South East	2611	107	41.5 (39.1- 43.9)
South West	3516	150	43.4 (40.9- 45.9)
South South	4345	132	30.4 (28.3- 32.5)
**Household wealth index**			
Poor	12542	499	39.3 (36.9- 41.7)
Middle	10013	355	35.6 (33.4- 37.8)
Rich	4592	142	31.6 (29.5- 33.5)
***Individual related factors***			
**Mother’s religion**			
Traditionalist and other	464	19	42.2 (39.8- 44.6)
Islam	15018	520	34.2 (32.0- 36.4)
Catholic and other Christian	11517	451	39.7 (37.3- 42.1)
Missing	148	5	
**Mother’s age at first birth**			
Less than 20 years	15755	598	37.6 (35.3- 39.9)
20 - 29 years	10702	363	34.2 (32.0- 36.4)
30 - 49 years*	690	35	52.8 (50.1- 55.3)
**Mother’s age**			
< 20	1471	92	65.0 (62.0- 68.0)
20-29	13096	451	34.5 (32.3- 36.7)
30-39	9797	327	33.1 (30.9- 35.3)
40-49	2783	126	44.7 (42.2- 47.2)
**Mother’s literacy level**			
Cannot read at all	15391	616	38.1 (35.8- 40.4)
Able to read	10634	370	34.1 (31.8- 36.2)
**Mother’s education**			
No education	12685	470	36.6 (34.3- 38.9)
Primary	6255	246	39.5 (37.1- 41.9)
Secondary or higher	8207	280	34.6 (32.4- 36.8)
**Mother’s desire for pregnancy**			
Wanted then	23880	824	34.7 (32.5-36.9)
Wanted later	1669	59	31.2 (31.0- 35.4)
Wanted no more	1150	43	38.3 (36.0- 40.6)
missing	448	71	
**Mother’s body mass index (BMI)**			
BMI > 18.5	23508	868	37.0 (34.7- 39.3)
BMI ≤ 18.5	3062	110	35.0 (32.8- 37.2)
Missing	577	18	
**Mother’s working status**			
Not working	9389	332	35.8 (33.5- 38.0)
Working	17121	638	37.1 (34.8- 39.4)
Missing	64	2	
**Sex of child**			
Female	13342	424	31.7 (29.6- 33.8)
Male	13806	572	41.4 (39.0- 43.8)
**Mother’s perceived baby size**			
Small or very small	3743	210	57.0 (54.2- 59.8)
Average or larger	22778	685	30.0 (27.9- 32.1)
Missing	626	102	
**Birth order and birth interval**			
First child	5331	249	47.6 (45.0- 50.2)
2 or 3 child, interval > 2	6444	160	24.3 (22.5- 26.2)
2 or 3 child, interval ≤ 2	2652	135	50.2 (47.5- 52.9)
4 or more child, interval > 2	9369	258	27.3 (25.3- 29.3)
4 or more child, interval ≤ 2	3352	194	57.6 (54.7- 60.5)
**Mode of delivery**			
Non-caesarean	26660	951	35.8 (33.5- 38.1)
Caesarean section	452	40	89.9 (86.3- 93.5)
Missing	35	5	
**Baby weight at birth**			
Less than 2500 grammes	349	16	46.6 (44.0- 49.2)
2500 - 3500 grammes	3110	45	14.7 (13.3- 16.1)
Greater than 3500 grammes	1458	15	9.2 (8.1- 10.3)
Not weighed	18923	767	40.7 (38.3- 43.1)
Missing	3307	153	
**Delivery assistance**			
Health professional	10331	381	37.0 (34.7- 39.3)
non-Health professional	16440	555	33.8 (31.6- 36.0)
Missing	377	61	
**Postnatal check-up**			
0 - 2 days	4372	112	26.1 (24.2- 28.0)
3 - 41 days	3001	78	26.0 (24.1- 27.9)
Don’t know/missing	19774	806	
**Birth place of child**			
Health facility	9917	361	36.8 (34.5- 39.1)
Home	16962	579	34.1 (31.9- 36.3)
Missing	268	56	
**Antenatal care**			
Yes	9421	232	24.6 (22.7- 26.5)
No	6316	169	26.5 (24.6- 28.4)
Missing	11411	595	

*Interval for 30–39 years and 40–49 years were merged.

NMR not calculated for missing values.

Neonates delivered by caesarean section had a higher NMR than those born vaginally (NMR: 89.9 vs 35.8). The NMR for male neonates was higher than that for female neonates (NMR: 41.4 vs 31.7).

### Multivariable analysis

Newborns born to mothers residing in rural areas had a higher risk of neonatal mortality than those who lived in urban areas (HR = 1.26, 95% CI: 1.03–1.55, p = 0.026). Compared with neonates born to mothers aged between 30 and 39 years, neonates born to younger mothers (<20 years) (HR = 4.07, 95% CI: 2.83–5.86, p < 0.001) reported a significantly higher risk of neonatal deaths. When the place of residence was replaced by household wealth index in the final model, neonates born to mothers in poor households had a high risk of neonatal death, although this was not statistically significant (HR = 1.24, 95% CI: 0.93–1.65).

Male neonates were more likely (HR = 1.30, 95% CI: 1.12–1.53, p = 0.001) to die than female neonates in the first month of life. Neonates delivered by caesarean section had a significantly higher risk of neonatal mortality (HR = 2.80, 95% CI: 1.84–4.25, p <0.001) compared with non-caesarean delivery. Neonates whose birth size were perceived by their mothers as small or smaller were also more likely to die than those of average or larger-sized neonates (HR = 2.10, 95% CI: 1.77–2.50, p < 0.001).

As shown in Table 3, there was a significantly higher risk of neonatal death for fourth or higher birth order neonates with a short birth interval ≤ 2 years (HR = 2.19, 95% CI: 1.68–2.84, p < 0.001), second or third birth order neonates with a short birth interval ≤2 years (HR = 1.75, 95% CI: 1.31–2.34, p < 0.001), and fourth or higher birth order with a longer birth interval > 2 years (HR = 1.36, 95% CI: 1.05–1.78, p = 0.022) compared with second or third birth order neonates with a longer birth interval > 2 years.

**Table 3:** Adjusted and unadjusted hazard ratios (95% confidence interval [CI]) for variables associated with neonatal mortality

**Variables**	**Unadjusted**			**Adjusted**^**^**^		
	**HR**	**[95% CI]**	***P***	**HR**	**[95% CI]**	***P***
***Community level factors***						
**Residence type**						
Urban	1.00			1.00		
Rural	1.36	(1.11-1.66)	0.003	1.26	(1.03-1.55)	0.026
**Geopolitical zone**						
North Central	1.00					
North East	1.23	(0.98-1.55)	0.072			
North West	1.07	(0.85-1.37)	0.540			
South East	1.12	(0.79-1.59)	0.536			
South West	1.28	(0.97-1.68)	0.079			
South South	0.82	(0.59-1.14)	0.235			
**Household wealth index**						
Poor	1.45	(1.11-1.89)	0.006			
Middle	1.20	(0.91-1.59)	0.189			
Rich	1.00					
***Individual related factors***						
**Mother’s religion**						
Traditionalist and other	1.00					
Islam	0.80	(0.48-1.31)	0.370			
Catholic and other Christian	0.88	(0.53-1.45)	0.605			
**Mother’s age at first birth**						
Less than 20 years	1.00					
20 - 29 years	0.88	(0.75-1.04)	0.127			
30 - 49 years*	1.34	(0.85-2.13)	0.209			
**Mother’s age**						
< 20	4.02	(2.99-5.40)	< 0.001	4.07	(2.83-5.86)	< 0.001
20-29	1.12	(0.93-1.33)	0.226	1.22	(1.00-1.49)	0.056
30-39	1.00			1.00		
40-49	1.34	(1.04-1.71)	0.023	1.27	(0.99-1.63)	0.063
**Mother’s education**						
No education	1.17	(0.96-1.43)	0.138			
Primary	1.20	(0.95-1.51)	0.168			
Secondary or higher	1.00					
**Mother’s literacy level**						
Cannot read at all	1.23	(1.03-1.46)	0.020			
Able to read	1.00					
**Mother’s desire for pregnancy**						
Wanted then	1.00					
Wanted later	1.07	(0.78-1.48)	0.670			
Wanted no more	1.17	(0.78-1.76)	0.442			
***Individual related factors***						
**Mother’s body mass index**						
BMI > 18.5	1.00					
BMI ≥ 18.5	1.08	(0.84-1.38)	0.566			
Missing						
**Mother’s working status**						
Not working	1.00					
Working	0.97	(0.83-1.15)	0.749			
Missing						
**Sex of child**						
Female	1.00			1.00		
Male	1.29	(1.11-1.51)	0.001	1.30	(1.12-1.53)	0.001
**Mother’s perceived baby size**						
Small or very small	2.17	(1.82-2.58)	< 0.001	2.10	(1.77-2.50)	< 0.001
Average or larger	1.00			1.00		
**Birth order and birth interval**						
First child	1.77	(1.38-2.25)	< 0.001	1.32	(1.01-1.72)	0.040
2 or 3 child, interval > 2	1.00			1.00		
2 or 3 child, interval ≤ 2	1.78	(1.34-2.38)	< 0.001	1.75	(1.31-2.34)	< 0.001
4 or more child, interval > 2	1.28	(1.01-1.62)	0.039	1.36	(1.05-1.78)	0.022
4 or more child, interval ≤ 2	2.10	(1.64-2.70)	< 0.001	2.19	(1.68-2.84)	< 0.001
**Mode of delivery**						
Non-caesarean	1.00			1.00		
Caesarean section‴	2.33	(1.54-3.51)	< 0.001	2.80	(1.84-4.25)	< 0.001
**Delivery assistance**						
Health professional	1.00					
non-Health professional	1.99	(0.84-1.17)	0.924			
**Birth place of child**						
Health facility	1.00					
Home	1.00	(0.84-1.17)	0.955			

‴Caesarean section is a combination of both elective and emergency caesarean.

*Interval for 30–39 years and 40–49 years were merged.

^^^2,465 missing information were not included in the analysis.

HR, hazard ratio; P-values based on Cox regression.

## Discussion

The overall aim of this study was to identify risk factors associated with neonatal mortality in Nigeria using a nationally representative sample. This study showed several factors that were significantly associated with neonatal mortality after adjusting for confounding factors, and each of these factors are discussed below.

We found that the NMR for singleton live-born infants between 2003 and 2008 was 36.7 (95% CI: 34.4–39.0). However, a preliminary report from the 2013 NDHS indicated that the NMR slightly fell by approximately 8% from 40 deaths per 1000 live births in 2008 to 37 in 2013 [23]. Despite this decline, Nigeria still has a long way to go in achieving the Millennium Development Goal 4 target for the under-5 years of age mortality rate.

Our study showed that male neonates had a significantly higher risk of dying during the neonatal period compared with female neonates. This finding is consistent with a cross-sectional study conducted in Indonesia in 2008, which indicated that male neonates were more likely to die than female neonates [19]. Additionally, a cross-sectional study performed in Bangladesh in 2009 reported a lower relative risk for female neonates compared with male neonates [24]. An increased risk of dying in the first month of life among male neonates may be attributed to high vulnerability to infectious disease [25]. Another possible reason for the low rate of neonatal deaths among girls may be because of the development of early fetal lung maturity in the first week of life [26], resulting in a lower incidence of respiratory diseases in female neonates compared with male neonates. Globally, it is estimated that approximately 23% of newborn deaths are attributed to respiratory problems [27].

In our study, mothers who perceived the size of their newborns to be small or very small had a 2.26 times greater risk of dying in the first month of life than those mothers who perceived their neonates to be of average or larger size. Similarly, findings from a cross-sectional study conducted in five Asian countries (India, Indonesia, Nepal, Bangladesh, and the Philippines) in 2008 also showed that smaller than average neonates had an increased risk of neonatal deaths than average or larger sized neonates in four of the five countries with data on perceived newborn size [28]. Even though our finding on perceived size of newborns were significant, we need to exercise caution in interpreting this result because the rationale mothers used in estimating the size of their neonates is unclear. However, this measure is not an unreasonable proxy for birth weight because a previous study showed a correlation between perception of birth weight and actual birth weight [21].

Our study showed that neonates delivered by caesarean section had a higher relative risk of neonatal mortality compared with vaginal deliveries. This result contradicts previous reports, which indicated a statistically insignificant relationship between the mode of delivery and neonatal mortality [29]. A similar study conducted in Swaziland reported a higher risk of death for neonates delivered by caesarean section than vaginal delivery, but this was not significant [30]. The significantly high risk of caesarean section observed in our study may be attributed to negative perceptions, such as misconception, fear, and aversion to caesarean section among mothers in Nigeria [31,32]. This could explain why pregnant mothers are presented to health facilities after experiencing labor at home or elsewhere, with life-threatening complications for emergency caesarean section [33]. This is also supported by a recent study on caesarean section and perinatal mortality in South Western, Nigeria, which found that nearly 84% of early neonatal deaths occurred in pregnant mothers who delivered their newborns by emergency caesarean section [34].

The current study observed that neonates born to mothers aged younger than 20 years had a significantly higher risk of mortality than those born to mothers aged 20–29 years, 30–39 years, and 40–49 years. This finding is similar to that reported in previous studies [35,36]. However, our analysis is not consistent with cross-sectional studies conducted in Swaziland and Tanzania, which found no significant relationship between maternal age and neonatal mortality [30,37]. This significantly higher rate of death in Nigeria could be related to inadequate use of maternal health services, physical immaturity, poor nutritional status, inexperience regarding child rearing among younger mothers, and poor maternal health outcomes, such as pregnancy complications. These factors are more common in younger mothers, and are all possible factors that could lead to higher adverse effects of neonatal and child health outcome in young motherhood [35].

We found that children of birth order (2 through 4 or higher) born with a shorter birth interval (≤2 years) were at higher risk of dying than those with a longer birth interval (>2 years). This finding is similar to a cross-sectional study carried out in India, which showed that neonates of fourth or higher birth order with a shorter birth interval of < 2 years have an increased risk of death compared with those of second and third birth order with a longer birth interval of > 2 years [38]. Maternal depletion syndrome could be attributed to this finding. Short-interval births could have adverse effects on the mother’s biological well-being and there could be economic resource competition between infants, especially in poor households, as well as inadequate care given to infants compared with high-ranked infants [39].

The current study showed that neonates born to mothers residing in rural areas had a higher risk of neonatal mortality compared with those living in urban areas. This finding is consistent with previous studies [40,41], which attributed this finding to limited access to health facilities and maternal healthcare services, such as delivery assisted by a healthcare professional, and prenatal and postnatal care. This disproportionally hinders rural dwellers from receiving adequate healthcare services, resulting in a high probability of neonatal death. In Nigeria, as in many developing countries, the majority of well-equipped hospitals and health centers are typically located in urban areas. However, neonatal jaundice and sepsis, as well as gestational age, which were previously found to be significantly associated with neonatal morality in most hospital-based studies [42,43], were not examined in our study. These variables could potentially be determinants of neonatal mortality in Nigeria.

The strengths and weaknesses of this study need to be considered when drawing specific inferences. This study was a nationally representative survey, with a stratified two-stage cluster sampling design, which achieved a 98.3% response rate. Additionally, recall errors arising from dates of birth and death given by women interviewed in the survey were minimized by restricting our analyses to births within the 5-year period preceding the survey. Third, the proportion of missing data was relatively small, such that it may not have influenced findings in our study. Despite these strengths, a number of weaknesses were also present in the study and they are as follows. (1) Only surviving women were interviewed, which may have led to under-reporting of the number of newborn deaths because of the association of neonatal death with maternal death [22]. (2) Gestational age, which may be an important risk factor for neonatal mortality, was not examined in this study. (3) Other factors previously found to be associated with neonatal mortality, such as antenatal care, postnatal care, and birth weight at birth, were lacking in information in the 2008 NDHS. (4) The Demographic and Health Surveys are the largest source of national data, but they are expensive and time consuming, and in Nigeria, this survey is usually conducted once in every 5 years. (5) Causal effects could not be measured because the study was based on a retrospective cross-sectional study.

A community-based interventional study on reducing neonatal death in Nigeria should be performed to focus on using verbal autopsy and birth weight. To reduce the recall period of using these instruments, a verbal autopsy should be undertaken before the culturally prescribed mourning period [44]. In addition, traditional birth attendants should be provided training or refresher training on delivery, how to recognise signs of pregnancy complications, and how to measure the newborn’s weight at birth because approximately 62% of mothers in Nigeria deliver their newborns at home [7].

## Conclusions

Our analysis of factors associated with neonatal mortality in Nigeria revealed that living in rural areas, child bearing at a younger age, birth order and birth interval, sex of the newborn (being male), caesarean delivery, and mothers who perceive their newborns as smaller than average at birth significantly increased the risk of neonatal death. Our findings indicate the need to implement community based newborn care interventions particularly, educating community health workers and traditional birth attendants about safe delivery practice, the benefits of Kangaroo mother care method on low birth weight newborns, child spacing and promote delay of first pregnancy will contribute to the improvement of neonatal mortality statistics in Nigeria.

## Competing interests

The authors declare that they have no competing interests.

## Authors’ contributions

OKE and KEA were involved in the conception and design of this study. OKE carried out the analysis and drafted the manuscript. KEA, MJD, JH, and ANP gave advice on interpretation and revised and edited the manuscript. All authors read and approved the final manuscript.

## Pre-publication history

The pre-publication history for this paper can be accessed here:

http://www.biomedcentral.com/1471-2458/14/521/prepub
